# Analysis of viral protein-2 encoding gene of avian encephalomyelitis virus from field specimens in Central Java region, Indonesia

**DOI:** 10.14202/vetworld.2016.25-31

**Published:** 2016-01-12

**Authors:** Aris Haryanto, Ratna Ermawati, Vera Wati, Sri Handayani Irianingsih, Nastiti Wijayanti

**Affiliations:** 1 Department of Biochemistry, Faculty of Veterinary Medicine, Universitas Gadjah Mada, Yogyakarta, Indonesia; 2 Division of Biotechnology, Animal Disease Investigation Center Wates, Daerah Istimewa Yogyakarta Province, Indonesia; 3 Division of Virology, Animal Disease Investigation Center Wates, Daerah Istimewa Yogyakarta Province, Indonesia; 4 Department of Animal Physiology, Faculty of Biology, Universitas Gadjah Mada, Yogyakarta, Indonesia

**Keywords:** avian encephalomyelitis, reverse transcription polymerase chain reaction, viral protein 2 gene

## Abstract

**Aim:**

Avian encephalomyelitis (AE) is a viral disease which can infect various types of poultry, especially chicken. In Indonesia, the incidence of AE infection in chicken has been reported since 2009, the AE incidence tends to increase from year to year. The objective of this study was to analyze viral protein 2 (VP-2) encoding gene of AE virus (AEV) from various species of birds in field specimen by reverse transcription polymerase chain reaction (RT-PCR) amplification using specific nucleotides primer for confirmation of AE diagnosis.

**Materials and Methods:**

A total of 13 AEV samples are isolated from various species of poultry which are serologically diagnosed infected by AEV from some areas in central Java, Indonesia. Research stage consists of virus samples collection from field specimens, extraction of AEV RNA, amplification of VP-2 protein encoding gene by RT-PCR, separation of RT-PCR product by agarose gel electrophoresis, DNA sequencing and data analysis.

**Results:**

Amplification products of the VP-2 encoding gene of AEV by RT-PCR methods of various types of poultry from field specimens showed a positive results on sample code 499/4/12 which generated DNA fragment in the size of 619 bp. Sensitivity test of RT-PCR amplification showed that the minimum concentration of RNA template is 127.75 ng/µl. The multiple alignments of DNA sequencing product indicated that positive sample with code 499/4/12 has 92% nucleotide homology compared with AEV with accession number AV1775/07 and 85% nucleotide homology with accession number ZCHP2/0912695 from Genbank database. Analysis of VP-2 gene sequence showed that it found 46 nucleotides difference between isolate 499/4/12 compared with accession number AV1775/07 and 93 nucleotides different with accession number ZCHP2/0912695.

**Conclusions:**

Analyses of the VP-2 encoding gene of AEV with RT-PCR method from 13 samples from field specimen generated the DNA fragment in the size of 619 bp from one sample with sample code 499/4/12. The sensitivity rate of RT-PCR is to amplify the VP-2 gene of AEV until 127.75 ng/µl of RNA template. Compared to Genbank databases, isolate 499/4/12 has 85% and 92% nucleotide homology.

## Introduction

Avian encephalomyelitis (AE) is a viral disease which can infect chickens, especially young chickens, birds, quails and turkeys [1-3]. The incidence of AE in Indonesia has been reported since 2009, namely in Lampung Province, Banten Province, Central Java Province, Yogyakarta Province and East Kalimantan Province on Indonesia. In general, the clinical symptoms in infected chickens are ataxia, incoordination, paralysis, and rapid tremor of the head and neck. Transmission of AE virus (AEV) infection generally occurs through infected eggs (egg transmission) and fecal-oral route [2]. AE incidence is always accompanied by a drastic decrease of egg production in laying hens and an increase in morbidity and mortality up to 80%. AEV infection may also increase the susceptibility of poultry to other infection agents, therefore, it is extremely harmful, especially in the commercial layer and broiler industry.

AEV causes disease in poultry worldwide, and flocks must be vaccinated for protection. Based on its protein composition, AEV is most closely related to those of hepatitis A virus (HAV) [4]. AEV is a member of the family Picornaviridae, which is classified into six genus, *Enterovirus, Rhinovirus, Cardiovirus, Aphthovirus, Hepatovirus*, and *Parechovirus* [5]. Member of the family Picornaviridae consists of small positive sense single-stranded RNA genome of more than 9.243 nucleotides excluding poly(A) tail, the kind of viruses which are capable of infecting various vertebrate species, including birds [6,7]. Chicken are an important reservoir for diverse picornaviruses that may cross avian species barriers through mutation and recombination [8]. AEV could be detected as a novel picornavirus in domesticated common quail (*Coturnix coturnix*) [9] while the novel turkey picornaviruses have been reported to be metagenomically detected, and the complete genome is characterized in fecal samples from healthy and affected turkeys in Hungary and USA [10,11].

Viral particles AEV has a diameter of 24-32 nm, non-enveloped with a sedimentation co-efficient of 148 Svenberg. AEV consists of 7.032 nucleotides, single sequence and open reading frame (ORF) that encodes a large polyprotein. It has four structural proteins in the region of P1 (VP-4, VP-2, VP-3, VP-1) and seven nonstructural proteins in P2 and P3 regions. Gene that encodes viral protein 2 (VP-2) in AEV is a more conserve and it does not have any similarity with VP-2 gene of other viruses [12]. Therefore, the use of a specific primer with VP-2 gene target is a more appropriate for diagnosis of AE disease than other genes. VP-1 protein is a major host-protective immunogenic against AEV challenge and demonstrates further that the antibody raised against VP-1 protein could neutralize AEV infection the more effective than antibody against the VP-3 or VP-0 protein in a virus neutralization (NV) test. Based on its sensitivity and specificity, it is indicated that VP-1 protein has a highly promising and reliable diagnostic potential and a suitable antigen for enzyme-linked immunosorbent assay (ELISA) detection of AEV antibodies in chickens [13].

In the field, the diagnosis of AE is still quite difficult, because a lot of poultry disease have very similar clinical symptoms, such as Newcastle Disease (ND), Marek’s Disease, Ricketsia, deficiency of B1 and B2 vitamins, *Aspergillosis, Salmonellosis, Coccidiosis, Omphalitis*, and *Mycoplasmosis*. Diagnosis of the disease is still confusing, and the serological tests do not show any difference between various kinds of AEV isolates [2]. A variety of diagnostic methods has been developed to diagnose AEV, which include AEV isolation using intracerebral inoculation into day old chicken, inoculation and propagation into embryonated fowl eggs (yolk sac). The serological diagnosis has also been developed such as hemagglutination (HA) test, complement fixation (CF), indirect fluorescence antibody, ELISA, VN, and agar gel precipitation (AGP) [14]. Serological test by ELISA for detection of antibodies against AEV in the chicken serum is prone to errors due to blood samples mishandling, such as heat treatment, repetitive freezing and thawing and hemolysis severity so it can affect ELISA results [15]. In commonly occurring and economically influential poultry disease, such as infectious bronchitis (IB), chicken anemia (CA) and AE, the commercially available ELISAs are routinely used as diagnostic tools to determine whether an individual bird or a flock has been previously exposed to IBV, CAV, or AEV [16]. Besides that, a rapid non-radioactive digoxigenin DNA probe is used to detect AEV. This probe hybridized specially with a DNA fragment of VP-1 gene with the sensitivity rate as little as 10 pg of targeted DNA fragment [17]. However, these diagnostic methods still have weakness and deficiencies, namely requiring a long time and skilled laboratory personnel, cross-reaction, false positive or false negative, less sensitive and expensive.

The routine AEV diagnosis which is performed in the laboratory is serological tests, by using HA test, HI test, and CF test (CFT) to detect the presence of antibodies against the hemagglutinin protein. Another laboratory test is AGP test. Serological methods are less effective in poultry because poultry is often infected by other viruses which have the same protein. Some viruses are often found to have some serotypes and genotypes so that the sensitivity and specificity of these tests are low [18]. Another diagnostic method is routinely performed by viral isolation from embryonated chicken eggs.

Although serological test and virus isolation from embryonated chicken egg have a high specificity and sensitivity, they have some disadvantages, such as taking long time, requiring modern laboratory facilities, requiring skilled human resources, and high cost. Molecular-based diagnostic method has some advantages in terms of rapidly, sensitivity and specificity to diagnose viral diseases [19].

The objective of this research was to analyze VP-2 encoding gene of AEV from various species of birds in field specimen by reverse transcription polymerase chain reaction (RT-PCR) amplification using specific nucleotides primer for confirmation of AE diagnosis. By this molecular method, it was expected to detect AEV rapidly, accurately and sensitively. It could be confirmed the differential diagnosis of AE in the field specimen and commercially poultry industry, it could be used as a basic study for viral disease surveillance and molecular epidemiology study in poultry diseases.

## Materials and Methods

### Ethical approval

AEV isolates were propagated and approved by the ethical committee in Animal Disease Investigation Center (ADIC) in Wates, Yogyakarta. All laboratory protocols and research procedures were supervised and approved by the ethical committee in Laboratorium Penelitian dan Pengujian Terpadu (LPPT), Universitas Gadjah Mada, Yogyakarta, Indonesia.

### Virus samples

The virus samples were collected from field specimens by ADIC in Wates, Yogyakarta, Indonesia during the period 2011-2012. AEV were isolated from brain organ of chickens showing neurologic symptoms such as tremor and ataxia. They were inoculated and propagated into the yolk sac of 9-11 days old specific antibody negative embryonated chicken eggs. After 3-5 days post-inoculation, the embryos were removed from eggs. The embryo brains were harvested and collected into phosphate buffered saline (PBS) pH. 7.2, then they were homogenized by a sterile homogenizer. The homogenized brain tissues were then centrifuged at 14.000 rpm for 1 h. The supernatant was collected as a viral suspension for viral RNA extracting. The list of AEV isolates are presented in Table-1.

**Table-1:** The AEV isolates from field specimens which were collected from several district in Indonesia.

Sample number	Sample code	Type of bird	Area in Indonesia
1	1341/8/11	Layer	Ambarawa, Semarang
2	1340/8/11	Layer	Tengaran, Semarang
3	17/I/12 (P. 03/I/12)	Layer	Mlati, Sleman
4	1785/X/11	Layer	Karanganyar, Karanganyar
5	499/4/12	Layer	Danurejan, Yogyakarta
6	1443/09/2012	Duck	Sanden, Bantul
7	1428/09/2012	Duck	Gatak, Sukoharjo
8	1132/07/2012	Broiler	Ngaglik, Sleman
9	1019/06/2012	Layer	Karanglewas, Banyumas
10	1018/06/2012	Layer	Laweyan, Surakarta
11	958/06/2012	Layer	Jatinegara, Tegal
12	922/06/2012	Layer	Purwokerto Timur, Banyumas
13	921/06/2012	Layer	Jurang, Temanggung

AEV=Avian encephalomyelitis virus

### Research materials and primer for RT-PCR

The main research materials of this study consists of High Pure Viral Nucleic Acid Kit (cat. no: 11-858-874-001) from Roche, SuperScript^™^ III One-Step RT-PCR with Platinum Taq (cat. no: 12574-026) from Invitrogen, UltraPure^™^ agarose gel from (cat. no:16500-100) from Invitrogen, 1x Buffer Tris base-Boric acid-EDTA, PBS, RedSafe nucleic acid dye from Intron, aquabidest, distilled water, Blue Loading dye (cat. no: 10816-015), 1 kb DNA Ladder (cat. no: 29,278,801) from Promega, and the specific oligonucleotide primers (Table-2).

**Table-2:** Primer used for amplification of VP-2 gene of AEV [20].

Gene target	Primer sequence	RT-PCR product	Temperature
VP-2 gene	AE-1: 5’ -CTTATGCTGGCCCTGATCGT-3’ AE-2: 5’ -TCCCAAATCCACAAACCTAGCC-3’	619 bp	57°C 61°C

VP-2=Viral protein 2, RT-PCR=Reverse transcription polymerase chain reaction, AEV=Avian encephalomyelitis virus

### Viral RNA extraction

Extraction of AEV RNA was performed by High Pure Viral Nucleic Acid Kit based on standard procedures from Roche. A total of 200 µl viral suspension was extracted in 50 µl end volume of viral RNA.

### Amplification of VP-2 gene of AEV by RT-PCR

Amplification of VP-2 gene of AEV was performed by SuperScript^™^ III One-Step RT-PCR with Platinum Taq-based on the standard procedure from Invitrogen. RT-PCR amplification which begins by a single cycle of RT at 50°C for 30 min, followed by a pre-denaturation step at 94°C for 2 min. PCR amplification stages consist of three stages: Denaturation at 94°C for 15 s, annealing at 57°C for 30 s, and extension at 68°C for 45 s. Stages of PCR amplification is done repeatedly in 40 cycles. Amplification process was ended by a final extension at 68°C for 5 min. AE commercial live vaccine is used as a positive control in this amplification, whereas the negative control derived from sterile dH_2_O contains no virus.

### Electrophoresis of RT-PCR products

RT-PCR products were electrophoresed in 1.5% agarose gel (Invitrogen) with RedSafe dye (Intron) staining. Fragments DNA were visualized in the dark room by UV-Transilluminator. The positive result of RT-PCR product from VP-2 gene was indicated as a DNA fragment in the size of 619 bp.

### Sensitivity test of RT-PCR

RNA of sample code 499/4/12 was used as template for RT-PCR amplification. Initial RNA concentration of 1022 ng/µl then was adjusted by serial dilution as many as 7 times with the concentration of 1022 ng/µl, 511 ng/µl, 255.5 ng/µl, 127.75 ng/µl, 63.88 ng/µl, 31.94 ng/µl, and 15,97 ng/µl. Then, to determine the sensitivity of RT-PCR, each serial dilution of RNA was used as template for RT-PCR amplification with amplification condition similar to amplification condition for the VP-2 gene of AEV.

### DNA sequencing

DNA sequencing to determine the nucleotide sequence of VP-2 gene was carried out in Genetika Science Indonesia Co., by chain termination method (Sanger Method). Then, the DNA sequencing products were analyzed in multiple alignments and compared by the sequence of VP-2 gene of AEV database from Genbank by MEGA 5.05 software program.

## Results

**Figure-1:**
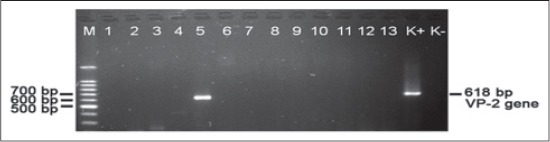
Electrophoresis of avian encephalomyelitis products of viral protein-2 gene from avian encephalomyelitis virus in agarose gel 1.5%. Fragments DNA of amplification products has the size of 619 bp. M is DNA ladder 100 bp, lane 1-13 are samples, K+ is positive control and K− is negative control. Sample No. 5 (sample code 499/4/12) is positive sample.

In this work, the analysis of VP-2 gene from AEV was conducted using RT-PCR. The amplification product was a fragment DNA in size of 619 bp. Electrophoresis of DNA fragments from RT-PCR products was presented in Figure-1.

The RT-PCR technique provides a faster and sensitive detection of AEV that might require some days and consecutive passages in cell culture for virus isolation. The application of RT-PCR method in molecular virology research for diagnosis of viral disease is expected to be more widespread. Then, the all 13 virus samples were also analyzed by serological test for detection of avian influenza (AI), ND viruses and molecular tested. The recapitulation of the serological and molecular test was presented in Table-3.

**Table-3:** Serological and molecular tests of 13 AEV isolates from field specimens.

Number	Sample code	Serological test	Molecular test (RT-PCR)
1	1341/8/11	AI (+), ND (−)	NT
2	1340/8/11	AI (+), ND (−)	NT
3	17/I/12, (P. 03/I/12)	AI, ND (−)	NT
4	1785/X/11	AI, ND (−)	NT
5	499/4/12	AI, ND (−), AE Suspect	AE (+)
6	1443/09/2012	AI (+), ND (−)	AE, ND (−), H5 (+)
7	1428/09/2012	AI (+), ND (−)	AE (−), ND, H5 (+)
8	1132/07/2012	AI, ND (−)	AE, AI (−)
9	1019/06/2012	AI, ND (−)	AE, AI (−)
10	1018/06/2012	AI, ND (−)	AE (−)
11	958/06/2012	AI, ND (−)	AE (−)
12	922/06/2012	AI (+), ND (−)	AE (−)
13	921/06/2012	AI, ND (−)	AE (−)

NT=Not tested, +=Positive result, -=Negative result, AI=Avian influenza, RT-PCR=Reverse transcription polymerase chain reaction, AEV=Avian encephalomyelitis virus

**Figure-2:**
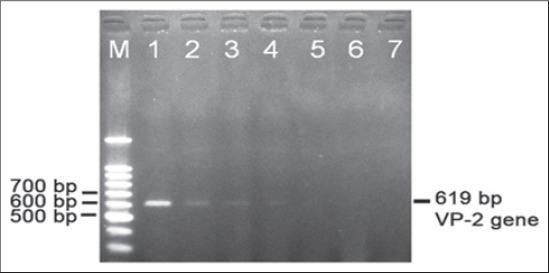
The sensitivity of RT-PCR method for detecting VP-2 gene of avian encephalomyelitis virus using RNA template from serial dilution of sample code 499/4/12 in agarose gel 1.5%. Lane M is marker DNA Ladder, lane 1-7 are number of serial dilution of RNA template with the concentration 1=1022 ng/µl, 2=511 ng/µl, 3=255.5 ng/µl, 4=127.75 ng/µl, 5=63.5 ng/µl, 6=31.94 ng/µl, and 7=15.97 ng/µl respectively.

Sensitivity test to the RT-PCR methods was conducted by serial dilution of sample no. 5 with the sample code 499/4/12 by the initial RNA concentration of 1022 ng/µl. The serial dilution was performed 7 times with the concentration of 1022 ng/µl, 511 ng/µl, 255.5 ng/µl, 127.75 ng/µl, 63.88 ng/µl, 31.94 ng/µl, 15.97 ng/µl. The RNA of serial dilution with this certain concentration then was used as a template for RT-PCR amplification. Amplification results using RNA template from serial dilution to determine the sensitivity test of sample code 499/4/12 was shown in Figure-2.

**Figure-3:**
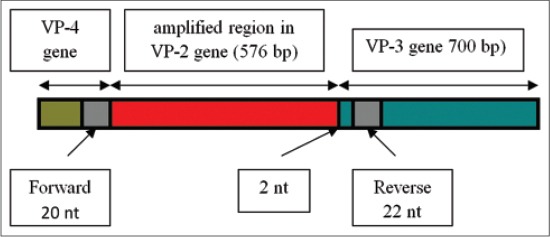
Schema of primers annealing and amplified region in VP-2 encoding gene of AEV.

Forward primer consists of 22 nucleotides and reverse primer consists of 20 nucleotides. The length of RT-PCR products was 619 bp, it is in accordance with the findings of the previous Xie *et al*. [20]. Location of forward primer annealing is into the VP-4 protein encoding gene region, while the location of reverse primer annealing is into the VP-3 protein encoding gene. Scheme of primers annealing and amplified region is presented in Figure-3.

## Discussion

Genome of AEV consists of around 7.5 kb with a single ORF encoding of 2.143 amino acids. As the other picornavirus, the ORF of AEV is divided into three regions, namely P1, P2 and P3 region. P1 region, especially encodes VP, VP-1, VP-2, VP-3 and VP-4 [21,22]. VP-1, VP-2, VP-3 and VP-4 proteins of AEV have a molecular weight in size of 43 kDa, 35 kDa, 33 kDa and 14 kDa respectively [1]. In this study, we analyze of VP-2 encoding gene of AEV by RT-PCR method using a specific pairs of oligonucleotide primer which were designed previously by Xie *et al.*, [20]. From 13 virus isolate samples of field specimens, a sample in lane 5 (sample code 499/4/12) is positive sample with clear DNA fragment of RT-PCR product in size of 619 bp, whereas 12 others samples from field specimens have no DNA fragment of RT-PCR product. Perhaps, these samples are not infected by AEV but infected by other avian pathogens or infectious agents, which have similar clinical symptom with AE. In lane K+ (positive control) also indicated DNA fragment in size 619 bp, this positive control originates from live vaccine AEV (CEVA). In lane K− (negative control) it is shown that there is no amplification product of VP-2 gene. Amplification products of the VP-2 encoding gene of AEV by RT-PCR methods of various type of poultry from field specimens showed a positive results on sample code 499/4/12 which generated DNA fragment in the size of 619 bp. This amplification results was in accordance with Xie *et al*. [20] who stated that RT-PCR method can detect AEV in more simple, sensitive and specific ways compared to AEV isolation and various immunologic methods. The use of fecal specimen has also been performed by Farkas *et al*. [23], who collected swabs and litter extracts from chickens, domestic ducks, turkeys and Canadian geese to detect novel picornaviruses by RT-PCR amplification, Liao *et al*. [24] detected and completely sequenced a novel picornavirus from Peking ducks (*Anas platyrhynchos domesitica*). Liu *et al*. [25] developed the rapid detection and quantification for AEV by using an SYBR Green real-time RT-PCR method for amplify VP-1 gene. Compared to conventional RT-PCR this method was 100 times more sensitive. It could detect levels as low as 10 standard DNA copies of the AEV from SX strain, so it was effective for AEV diagnosis and surveillance.

In Table-3, a total of 13 field specimen samples were serologically and molecularly tested for possible infection by ND and AI viruses (AIVs). The recapitulation showed that some of the field specimens were negatively infected by ND virus but positively infected by AIV. Molecular test for AEV using ELISA showed that only sample code 499/4/12 is positively infected by AEV, whereas others 12 samples are negative and not infected by AEV.

Then using positive sample (sample code 499/4/12), we further tested the sensitivity of RT-PCR method using serial dilution RNA template for RT-PCR amplification. This sensitivity test using RT-PCR methods was performed by serial dilution of RNA template using sample code 499/4/12. The initial RNA concentration for serial dilution using sample code no 499/4/12 was 1022 ng/µl. The serial dilution was performed 7 times with the concentration of 1022 ng/µl, 511 ng/µl, 255.5 ng/µl, 127.75 ng/µl, 63.88 ng/µl, 31.94 ng/µl, 15.97 ng/µl respectively. The RNA of serial dilution with this certain concentration then was used as template for RT-PCR amplification. Amplification results using RNA template from serial dilution to determine the sensitivity test of sample code 499/4/12 was shown in Figure-2. In this study, the sensitivity of RT-PCR method is 127.75 ng/µl, while according to Haryanto *et al*. [26] was 10 pg/µl. This result difference may due to differences in the conditions of field sample purity, method difference of viral RNA extraction and different conditions for RT-PCR amplification.

Manoharan *et al*. [27] have conducted PCR sensitivity tests to assess of VP-1 gene of CA virus (CAV) using three different primer sets. This study proved that PCR method is a useful technique for CAV DNA detection in field specimen, this study also demonstrates that the field CAV detection limit could be extended further to the level of detecting subclinical infection due to the use of the most sensitive primer. RT-PCR method was also performed to directly amplify from field specimens of fusion (F) protein encoding genes of NDV [26], the matrix (M) and hemaglutinin (H5) protein encoding genes of AIV H5N1 subtype [28], and viral protein-2 (VP-2) encoding gene of infectoius bursal disease virus (IBDV) [29].

Based on the RT-PCR product for sensitivity test by serial dilution of RNA template of sample code 499/4/12 (Figure-3). The sensitivity of RT-PCR amplification could be obtained until 4 times serial dilution which produced as little as 127.75 ng/µl of RNA template. It showed that the sensitivity of RT-PCR for amplification of VP-2 gene of AEV by serial dilution of RNA sample as template was high. That means a small amount of RNA template which was extracted directly from field specimens could be well amplified and visualized as a clear DNA fragment in agarose gel.

In this study, we did not have field samples from young birds of less than 4 weeks, because in this collecting period, all collected field samples derived from adults birds with neurological symptoms. There is a marked age resistance to clinical signs in birds exposed after they are 2-3 weeks of age. The clinical signs in exposed young chicks to have a minimum incubation period of 10-11 days, at the same time, that VN antibodies can be detected in adult birds [2]. Infection of AEV in mature birds may experience a temporary drop in egg production (5-10%) but it did not develop to the neurological symptoms [30].

Specificity of nucleotide primers used in this work was shown by DNA sequencing products of positive sample which was then analyzed using NCBI BLAST program. This analysis showed that nucleotides primers for amplification of VP-2 gene of this work has 92% homology to the AEV isolate (accession number AV1775/0/), and 85% identical to the AEV strain (accession number ZCHP2/0912695). It is indicated that RT-PCR method is specific to identify AEV from field specimen. Welchman de *et al*. [31] reported that AEV infecting pheasants were distinct from AEV infecting chickens. RT-PCR amplification for detecting of AEV in a non-standard host species should be supported by the appearance of the minimal lesion in central nervous system. Apart from that, immunohistochemistry would have been a useful diagnostic aid to demonstrate AEV in pigeons [32].

Based on the multiple alignments of VP-2 gene of AEV (isolate 499/4/12) in comparing with Genbank database, it found 46 nucleotides difference between 499/4/12 isolate with AV 1775/07 isolate from Genbank, whereas with ZCHP2/0912695 isolate found 93 nucleotides difference. Using MEGA version 5.05 software program, it was shown that positive isolate (499/4/12) is really positive amplification of VP-2 protein encoding gene from AEV. It strengthened the highly homologous nucleotides composition of tested positive sample with Genbank databases.

The common problem for AEV diagnosis is less rapid and time-consuming. The diagnosis of AE in field cases was just based on anamnesis of infected chickens in a flock, histopathological changes, and specific clinical symptoms from the infected chickens. RT-PCR method has advantages compared to other diagnostic methods, in AE cases, amplification of VP-2 gene can detect and identify the presence of AEVes more rapidly and specificcally, especially for field specimen samples. The high specificity and sensitivity of RT-PCR method indicated that this method can be used to detect VP-2 gene of AEV from field specimens. Reagents which are used for RT-PCR amplification can be prepared easily and take less amount. Inoculation and propagation of AEV into embryonated chicken eggs takes a long time, whereas molecular technique such as RT-PCR can provide the result within a day. For diagnosis of AEV, the RT-PCR method is more effective, efficient and specific compared to others diagnostic tests. RT-PCR method targeting VP-2 gene of AEV has some advantages and it is a quantitative diagnostic method, so it can not be used to determine the quantity of infectious agents.

Proper diagnosis of AE disease should be done early and rapidly to prevent the spreading of AEV to poultry that has not been infected yet. Rapid, sensitive and specific method for laboratory diagnosis of AE disease in poultry is needed. Laboratory diagnosis for AEV was conducted by virus isolation into embryonated chicken eggs and various immunological methods which required a lot of money, complicated laboratory apparatus and consumed a lot of time. This study indicated that the RT-PCR method targeting VP-2 gene of AEV can be used to detect and identify AEV from field specimen rapidly, effectively and efficiently so it has provided a significant contribution in AEV detection and AE disease spread control.

Currently, laboratory diagnosis of AE disease is performed by AEV isolation and detection with various immunologic methods that are laborious, highly costly and time-consuming. A rapid, sensitive, and specific method to diagnose of AE disease is still required for diagnosis confirmation. Xie *et al*. [20] have developed an RT-PCR method for AEV detection which is simple to employ as well as sensitive and specific.

## Conclusion

Analyses of the VP-2 encoding gene of AEV with RT-PCR method from 13 samples from field specimen generated the DNA fragment in the size of 619 bp from one sample with sample code 499/4/12. The sensitivity rate of RT-PCR is to amplify the VP-2 gene of AEV until 127.75 ng/µl of RNA template. Compared to Genbank databases, isolate 499/4/12 has 85% and 92% nucleotide homology.

## Authors’ Contributions

AH was responsible for the overall stage of research (research preparation, execution, completing), RE did the RT-PCR amplification and phylogenetic analysis, VW did the serological and molecular test, SHI did the virus isolation and MDT quantification, NW drafted and revised the manuscript. All authors contributed to support the research datas, they read and approved the final manuscript.
